# In sickness and in health: The functional role of extracellular vesicles in physiology and pathology in vivo: Part II: Pathology

**DOI:** 10.1002/jev2.12190

**Published:** 2022-01-18

**Authors:** Abi G. Yates, Ryan C. Pink, Uta Erdbrügger, Pia R‐M. Siljander, Elizabeth R. Dellar, Paschalia Pantazi, Naveed Akbar, William R. Cooke, Manu Vatish, Emmanuel Dias‐Neto, Daniel C. Anthony, Yvonne Couch

**Affiliations:** ^1^ Department of Pharmacology University of Oxford Oxford UK; ^2^ School of Biomedical Sciences Faculty of Medicine University of Queensland St Lucia Australia; ^3^ Department of Biological and Medical Sciences Faculty of Health and Life Sciences Oxford Brookes University Oxford UK; ^4^ Department of Medicine, Division of Nephrology University of Virginia Charlottesville Virginia USA; ^5^ Molecular and Integrative Biosciences Research Programme Faculty of Biological and Environmental Sciences University of Helsinki Helsinki Finland; ^6^ Division of Cardiovascular Medicine, Radcliffe Department of Medicine University of Oxford Oxford UK; ^7^ Nuffield Department of Women's and Reproductive Health John Radcliffe Hospital, Headington Oxford UK; ^8^ Laboratory of Medical Genomics. A.C. Camargo Cancer Centre São Paulo Brazil; ^9^ Laboratory of Neurosciences (LIM‐27) Institute of Psychiatry São Paulo Medical School São Paulo Brazil; ^10^ Acute Stroke Programme ‐ Radcliffe Department of Medicine University of Oxford Oxford UK

**Keywords:** exosomes, extracellular vesicles, in vivo, microvesicles, pathology, physiology

## Abstract

It is clear from Part I of this series that extracellular vesicles (EVs) play a critical role in maintaining the homeostasis of most, if not all, normal physiological systems. However, the majority of our knowledge about EV signalling has come from studying them in disease. Indeed, EVs have consistently been associated with propagating disease pathophysiology. The analysis of EVs in biofluids, obtained in the clinic, has been an essential of the work to improve our understanding of their role in disease. However, to interfere with EV signalling for therapeutic gain, a more fundamental understanding of the mechanisms by which they contribute to pathogenic processes is required. Only by discovering how the EV populations in different biofluids change—size, number, and physicochemical composition—in clinical samples, may we then begin to unravel their functional roles in translational models in vitro and in vivo, which can then feedback to the clinic. In Part II of this review series, the functional role of EVs in pathology and disease will be discussed, with a focus on in vivo evidence and their potential to be used as both biomarkers and points of therapeutic intervention.

## 1 INTRODUCTION

**FIGURE 1:**
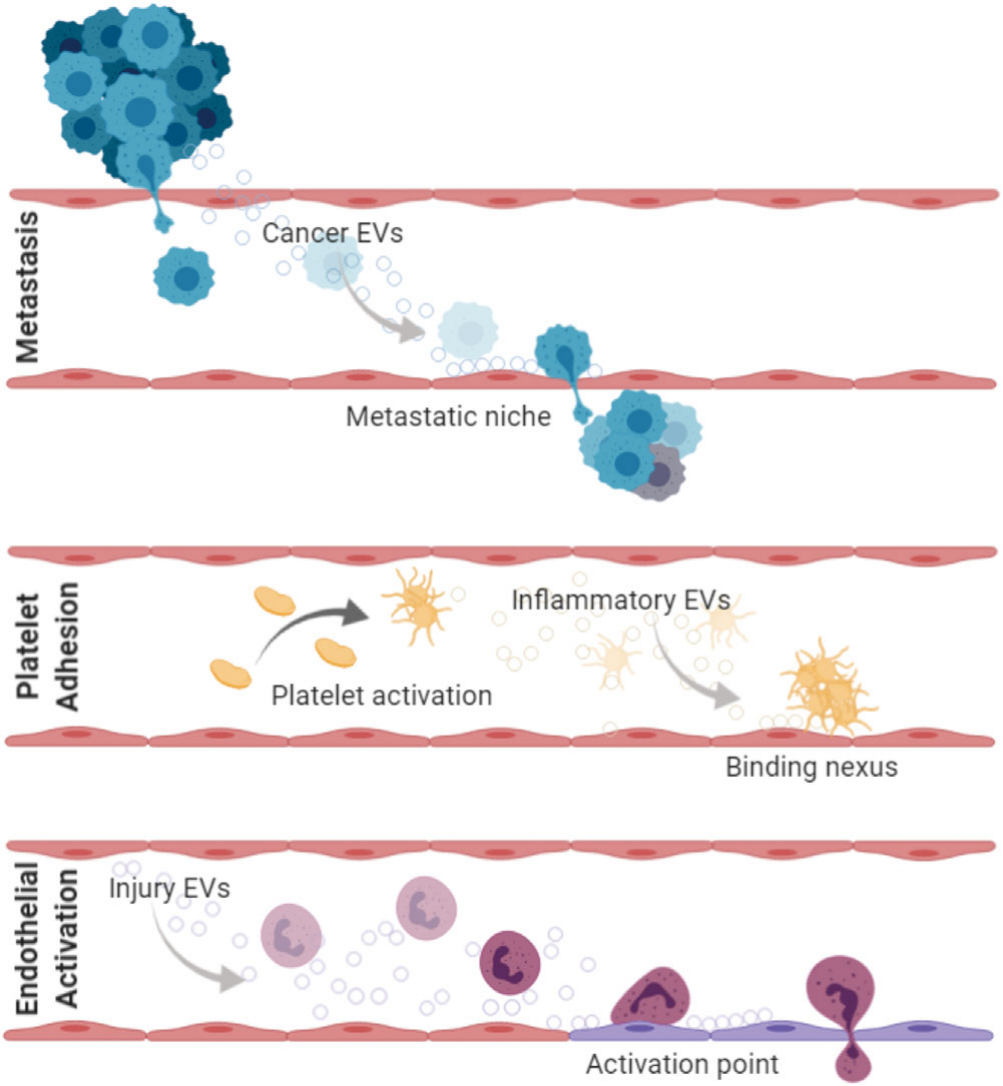
Examples of potential extracellular vesicle (EVs) interactions between different cell types and the vasculature. in vivo evidence suggests that EVs can act as a seed for the development of a metastatic niche (top panel), a binding nexus for platelet adhesion and activation (middle panel) and as an activation point for the endothelium in the inflammatory response (bottom panel). Taken from refs: (Agouni et al., 2019; Akbar et al., 2017; Anthony & Couch, 2014; Badimon et al., 2016; Becker et al., 2016; Cloutier et al., 2013; Costa‐Silva et al., 2015; Couch, Akbar, Davis et al., 2017; Couch, Akbar, Roodselaar et al., 2017; Deng et al., 2017; Dickens et al., 2017; Fabbri et al., 2012; Hazelton et al., 2018; Hoshino et al., 2015; Keklikoglou et al., 2019; Loyer et al., 2018; Maugeri et al., 2018; Meehan & Vella, 2016; Mörtberg et al., 2019; Motta‐Mejia et al., 2017; Peinado et al., 2012; Ridger et al., 2017; Siljander et al., 1996; Tominaga et al., 2015; Yates et al., 2019; H. Zhang, Deng, et al., 2017; L. Zhang et al., 2015; Zubairova et al., 2015)

**FIGURE 2:**
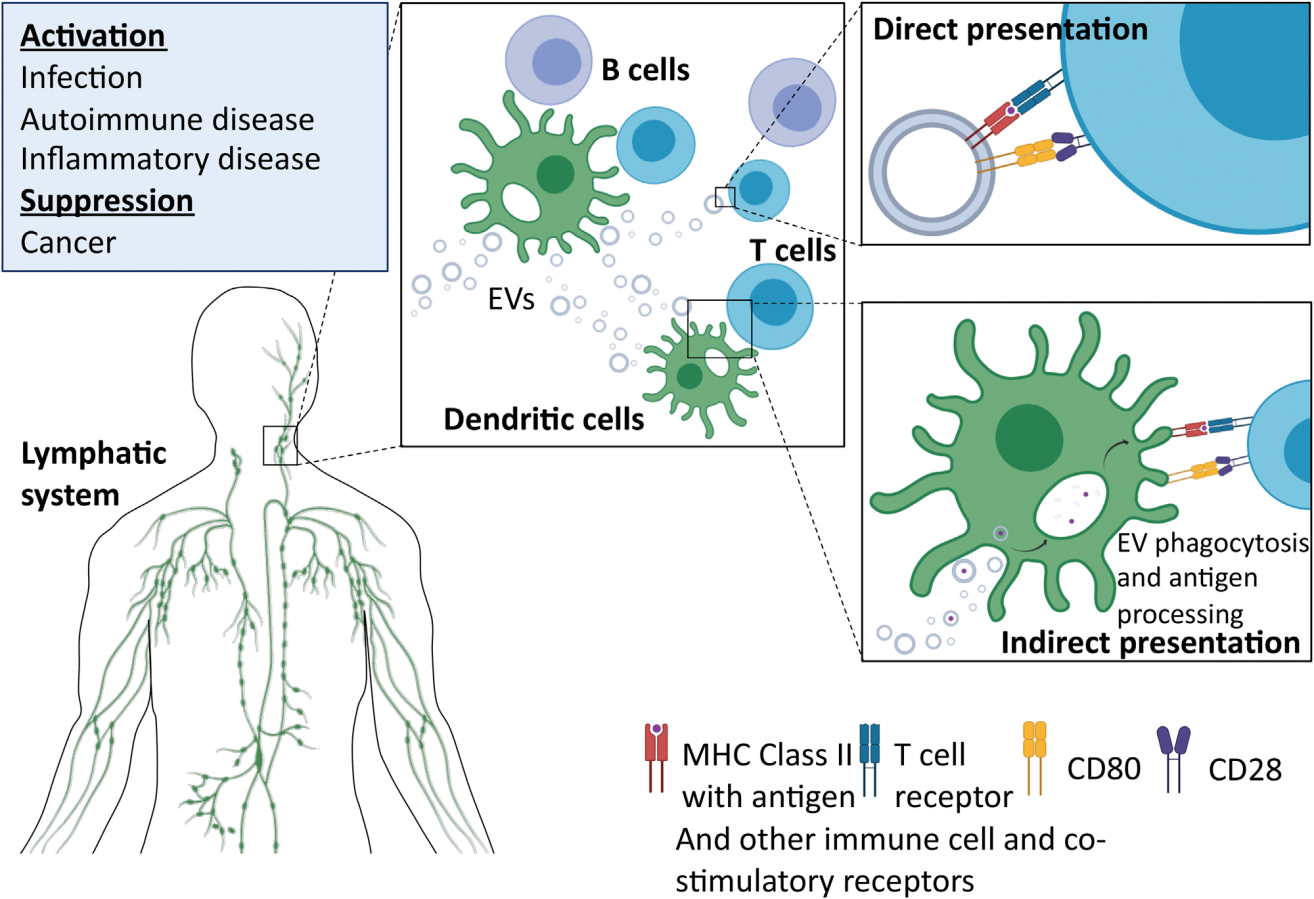
Examples of potential extracellular vesicle (EVs) interactions between cells of the immune system. EVs have been shown to play ambiguous roles in the transmission of initial infection by, for example, viruses (Dias et al., 2018). However, in terms of activation and suppression of the immune system there is considerable evidence from in vivo models of disease to suggest that EVs play a significant role. Taken from refs: (Akbar et al., 2017; Bashratyan et al., 2013; Blonda et al., 2018; Bobrie et al., 2012; Casella et al., 2018; Cloutier et al., 2013; Costa‐Silva et al., 2015; C. Liu et al., 2006; Dias et al., 2018; Hulsmans et al., 2013; Jansen et al., 2017; J.‐G. Wang et al., 2011; Kim et al., 2005; Kimura et al., 2018; Mitsuhashi et al., 2016; Rahman et al., 2014; Saenz‐Cuesta et al., 2014; Sheng et al., 2011; Ullal et al., 2011; Valenti et al., 2006)

Extracellular vesicles (EVs) play a pivotal role in intercellular communication. Whilst they have been shown to contribute to the maintenance of homeostasis (*see* Part I of this series), most research has focused on their involvement to disease. They can be enriched for pathogenic proteins and nucleic acids (Coleman & Hill, 2015; Fong et al., 2015), and have been shown to participate in the progression of a range of pathologies, including cardiovascular, renal and inflammatory diseases (discussed below). As such, there is growing interest in developing novel therapeutic strategies targeting EVs. However, our understanding of EV‐mediated signalling in disease pathophysiology is limited. Whilst there are significant numbers of papers using both clinical samples and animal models which characterize the EV population changes in disease, there are very few studies which directly associate these changes with downstream function. Determining the degree to which EVs are simply markers of disease, or active participants in the pathological processes of any given disease, will be key to effectively utilising them in the future (Figures 1 and 2). Given the heterogeneity of biofluid populations, and their widespread, systemic effects, only studying EVs in whole systems will improve our knowledge for therapeutic gain.

## 2 PLATELETS

Platelet biology falls at the interface of physiology and pathology. Whilst the process of coagulation can be considered a homeostatic response to injury, inappropriate platelet activation can result in pathological thrombosis (reviewed in Badimon et al., 2016), and thromboinflammatory diseases such as atherosclerosis (Figure 1). In early studies on haemostasis, it was recognised that platelet EVs account for approximately 25% of the procoagulant (and anticoagulant) activity in blood (Tans et al., 1991), and their surface chemistry exhibits a 50‐ to 100‐fold higher procoagulant activity than the surface of activated platelets (Sinauridze et al., 2007). By contrast, the lack of platelet EV formation is associated with increased and abnormal bleeding (reviewed in Melki et al., 2017). These observations highlight the importance of the expansion of the available catalytic surface by platelet EVs. The increased catalytic ability caused by the changes on the membrane surfaces between the parent platelets and the shed daughter EVs, is being exploited as a potential therapeutic strategy to improve haemostasis by generating artificial platelet vesicles (Girish et al., 2020).

In whole blood perfusion studies, fibrin fibres were shown to contain EV sized elements, positive for CD61, which is known to play a role in platelet aggregation, suggesting that platelet‐derived EVs bind and provide a procoagulant surface (Siljander et al., 1996). Therefore, platelet EVs may modulate fibrin formation kinetics, the structure of the clot, as well as fibrinolysis (Zubairova et al., 2015). In a rat model of haemorrhagic trauma, platelet EVs from human donors provided pro‐haemostatic support during uncontrolled bleeding and prevented haemorrhagic shock (Zubairova et al., 2015). Whilst this can be beneficial in the case of haemorrhage, platelet EVs generated after traumatic brain injury (TBI) were shown to play a key role in the development of a post‐traumatic hypercoagulable state in patients (Midura et al., 2015), which can lead to complications during recovery. Recent data demonstrated that platelet EVs transfer GPIbα to monocytes enabling them to be recruited to large and small blood vessels, an interaction that was initiated by P‐selectin and stabilized by phosphatidyl serine (Chimen et al., 2020). In trauma patients, monocytes bore platelet markers within 1 h of injury, the levels of which correlated with severity of trauma (Chimen et al., 2020), suggesting platelet EVs might be mediating the communication of vascular injury throughout the body.

In studies on inflammation, there are a number of possible roles for platelet EVs related to infection and sepsis (Kerris et al., 2020). For example, increased numbers of platelet EVs have been reported in response to H1N1 influenza virus and *Plasmodium* parasite infection (Kerris et al., 2020). Platelet EVs most likely participate in the immune response by modulating the performance of other immune cells, particularly neutrophils and monocytes, and so may contribute to the co‐ordination of the immune response. Certainly, in Dengue, EVs from virus‐activated platelets were shown to induce neutrophil extracellular trap (NET) formation and cytokine release from neutrophils and macrophages (Sung et al., 2019). This platelet EV‐mediated NET formation may also contribute to thrombosis, particularly in the venous side, which is a feature of Dengue viral infection. Thus, blocking the interaction between platelet EVs and leukocytes during infection improved survival from 30% to 90% in mice (Sung et al., 2019). The multifaceted interaction of platelets and neutrophils serves to illustrate the complexity and interlinkage of the EV‐mediated cell‐to‐cell communication (Ramirez et al., 2019). Platelet EVs have been shown to be internalised by neutrophils, via concerted actions of lipid modifying enzymes (Duchez et al., 2015). Neutrophils receive 12‐HETE from platelet EVs to synthesise leukotrienes via leukotriene 4 synthase, whilst neutrophil EVs deliver arachidonic acid to enable TXa2 production via COX1 in platelets. Recently, it was shown in two mouse models that the platelet EVs formed during inflammation (French et al., 2020) and acute liver injury infiltrated into the bone marrow and were essential for regulating megakaryopoiesis (Qu et al., 2020), and the infiltration was also validated in human bone marrow biopsies (French et al., 2020). Platelet EVs are likely to mediate many of their pathological functions by proxy, which hampers the identification of these roles in vivo.

Platelet EVs have also been implicated in autoimmunity, and their role is exemplified in rheumatoid arthritis (RA) (Tessandier et al., 2020). A seminal paper on the role of platelet EVs in RA showed IL‐1α and IL‐1β‐containing platelet EVs in the synovial fluid of RA patients (Boilard et al., 2010). In a mouse model of RA, the generation of these EVs was triggered by collagen receptor GPVI and these EVs promoted synoviocyte activation and production of IL‐6 and IL‐8/CINC‐1 (Boilard et al., 2010). Interestingly, platelet EVs also had the capacity to transduce peripheral blood‐derived regulatory T‐cells, preventing them from differentiating into IL‐17‐ and IFN‐γ‐producing proinflammatory cells (Dinkla et al., 2016). As these cells are reportedly present in RA (Ridger et al., 2017), the role of platelet EVs in this inflammatory disease may be multifaceted and highlights the importance of studying them in a system with multiple cell types.

In addition to inflammatory cells, platelet EVs also interact with the endothelium (Ridger et al., 2017). This interaction may underlie altered pathophysiology in various contexts, from atherosclerosis (Badimon et al., 2016) to brain function (Leiter & Walker, 2019) and angiogenesis (Doser et al., 2009). Systemic sclerosis patients were shown to have increased levels of platelet EVs, autophagic neutrophils and NET by‐products in their circulation (Maugeri et al., 2018). Studies in mice have demonstrated that HGMB‐1 on platelet‐EVs, in combination with the aforementioned immune activation, may provide the mechanistic underpinnings of this vasculopathy (Maugeri et al., 2018). Platelet EVs have been shown to be pro‐angiogenic in vivo (Mause et al., 2010; Todorova et al., 2017), mediating improvements in endothelial regeneration. Specifically, platelet EVs enhanced the vasoregenerative potential of early outgrowth cells after vascular injury in mice (Mause et al., 2010). Whilst the exact mechanisms of this angiogenic activity remain unclear, much has been attributed to the rich growth factor cargo of platelet EVs (Dean et al., 2009; Gaetani et al., 2018). In cases of vascular injury and disease this activity is obviously beneficial, however, angiogenesis during cancer is detrimental and therefore discovering the molecular pathways associated with these processes in vivo is key to exploiting them therapeutically.

It must be noted that the true in vivo role of platelet EVs, as opposed to that of platelets, is difficult to discern. In most cases, pathological states such as atherosclerosis have only been *associated* with changes in platelet EV concentration (Gasecka et al., 2019). Only rarely have the mechanisms or molecular determinants for the function of platelet EVs been identified, and, even then, predominantly in only in vitro studies. Since correlation does not indicate causality, the functional significance of platelet EVs in pathology is still unclear. Moreover, the most commonly used method to detect (platelet) EVs has been conventional flow cytometry, where the typical lower detection limit is well above 300 nm (Arraud et al., 2014). This will only detect <1%, and only the largest, (plasma) EVs. Therefore, the findings from many of the early platelet EV studies call for a revisit or verification by newer methods (Gasecka et al., 2019), such as EV‐adapted flow cytometry (Welsh et al., 2017), which may bring new insight to the field.

## 3 INFLAMMATION AND IMMUNITY

The field of EVs in immunology suffers somewhat from a chicken‐and‐egg issue. The naturally pro‐inflammatory environment present in patients with autoimmune or inflammatory disease is likely to precipitate an increase in circulating EVs. To what degree the circulating EVs are the original catalyst for disease, and to what degree they are a consequence, is yet unknown and highlights the importance of studying these processes in whole animals and patients where possible (Figure 2). An excellent example of this lack of physiological knowledge is viral infection. Genetic material from viruses such as the tick borne *Flaviviridae*, Hepatitis C virus and Epstein‐Barr virus have all been found in EVs (Dias et al., 2018) but the evidence for their capacity to spread beyond transfer between two isolated cell populations in vitro is lacking. Indeed, in their review Dias and colleagues suggest the evidence for the involvement of EVs in the infectivity and spread of diseases such as human immunodeficiency virus is ambiguous (Dias et al., 2018). Where cascade events occur, such as happens in the activation of the immune system, the interaction of multiple cell types and multiple signalling pathways is key to orchestration of the final outcome. Therefore, studying the role of EVs in these events is limited when using isolated cell populations.

### 3.1 Immune cell activation

In addition to acting as intercellular messengers between cells of the immune system, inappropriate EV‐mediated activation of the immune system can contribute to pathological conditions. An example from the previous section was that the presence of platelet‐derived EVs in the synovial fluid of RA patients is likely to be responsible for the formation of an inflammatory nexus within affected joints (Cloutier et al., 2013). In inflammatory bowel disease patients, granulocyte‐derived EVs enriched for metalloproteinases and pro‐inflammatory cytokines have been shown to increase gut permeability (Mitsuhashi et al., 2016). Additional studies have linked EV‐mediated inflammation to cardiovascular disease (CVDs) (Akbar et al., 2017; Amabile et al., 2014; Blonda et al., 2018; Hulsmans & Holvoet, 2013; Jansen et al., 2017; J.‐G. Wang et al., 2011), and multiple sclerosis (MS) (Blonda et al., 2018; Casella et al., 2018; Kimura et al., 2018; Sáenz‐Cuesta et al., 2014) among others. At the most basic autoimmune level, self‐antigen presentation to autoreactive lymphocytes by EVs can lead to activation of immune cells with antigen‐specificity for one's own cells, linking EVs to autoimmune diseases. Indeed, in systemic lupus erythematosus, antigenic DNA has been found in circulating vesicles which may act as an autoantigen to precipitate disease progression (Ullal et al., 2011). Moreover, EVs have been shown to trigger pancreatic inflammation and drive development of diabetes in NOD mice, by interacting with autoreactive T‐ and B‐cells directly (Bashratyan et al., 2013; Rahman et al., 2014; Sheng et al., 2011).

### 3.2 Immunosuppression and allergy

Pathologically, suppression of the immune system can be devastating. In cancer, EVs may act in the development of the metastatic niche, in particular there is a wealth of evidence to suggest they do this by suppressing the circulating immune response to migrating tumour cells (Costa‐Silva et al., 2015; C. Liu et al., 2006). An active inflammatory response to cellular outgrowth is a key mechanism for the reduction of tumour burden, one which is frequently exploited in modern tumour therapy, and EVs seem to play an important role in this process. Tumour EVs have been shown to activate regulatory T‐cells and Myeloid Derived Suppressor Cells (MDSCs), which inhibit CD8+ T‐cell mediated targeting of the tumour (Valenti et al., 2006). They have also been shown to express FasL and TRAIL which induce apoptosis of CD8+ T‐cells. Kim *et al*., found FasL+ microvesicles in the sera of 21/22 oral squamous cell carcinoma patients, levels of which correlated with tumour burden and nodal involvement (Kim et al., 2005). In a mouse model of metastatic cancer, C. Liu et al. (2006) showed that EVs suppress NK cell function. NK cells are known to have an important anti‐tumour cytotoxic response, so this suppression via EVs reduced the number of circulating cytotoxic NK cells in the lung and spleen, allowing metastatic niches to form in those organs. Further data has demonstrated that neutrophil mobilization is required for tumour growth, and that targeting EV release by GTPase RAB27A/B blockade decreased primary mammary carcinoma tumour growth and its dissemination into the lung (Bobrie et al., 2012).

## 4 THE CARDIOVASCULAR SYSTEM

Most of the work on cardiovascular EVs has been undertaken in pathology, hence the systems’ relatively minor position in Part I of this series. Indeed, plasma EVs have been shown to be biomarkers of CVDs (Alibhai et al., 2018; Amabile et al., 2014; Bellin et al., 2019; Gidlöf et al., 2019; Gollmann‐Tepeköylü et al., 2020; Jansen et al., 2017; Nozaki et al., 2009). Inflammatory CVDs, such as atherosclerosis, acute myocardial infarction and stroke, stem from endothelial cell activation and vascular dysfunction (Esper et al., 2006). Endothelial cells are uniquely placed in direct contact with the peripheral blood, which allows endothelial‐derived EVs to be rapidly released and undergo delivery to distant organs. Importantly, the endothelial cells of the vasculature form a surveillance system, they are amongst the first cells in the body to experience alterations in soluble blood‐borne factors, including elevations in plasma lipids, glucose, inflammatory proteins and perturbations in oxygen concentration. Therefore, their response, and their subsequent EV production, can act as a signal to changes in physiology or the onset of pathology.

As discussed in Part I, EVs have been shown to modulate blood pressure, which may be considered homeostatic, although they may also have downstream pathogenic consequences. For example, work in rats has identified that circulating EVs are capable of restraining vasodilation (Good et al., 2020; Otani et al., 2018), and that this may play an important role in hypertension. Indeed, increased endothelial EVs have been reported in the plasma (Preston et al., 2003) and urine (Sun et al., 2018) of hypertensive patients. Moreover, inhibition of EV generation and release reduced blood pressure in hypertensive rats (G. Zhang et al., 2020). Collectively, these data indicate that endothelial‐derived EVs contribute to vascular dysfunction. However, by contrast, the effects of endothelial cell‐derived EVs from injured tissues in the whole animal—e.g., from the heart following myocardial infarction—on vascular function, remains unknown. To date we know that circulating levels of endothelial cell‐derived EVs are elevated in patients with endothelial dysfunction (Leite et al., 2020), obstructive sleep apnoea (Jia et al., 2017), obesity (Esposito et al., 2006), renal failure (Amabile et al., 2005), coronary artery disease (Y. Liu et al., 2019), myocardial infarction (Loyer et al., 2018; Mörtberg et al., 2019), β‐thalassaemia (Kheansaard et al., 2018), and in stroke (Agouni et al., 2019) but our knowledge of the contribution of EVs—if they indeed contribute and are not simply epiphenomena—to these diseases, remains limited. As with the majority of EV work in vivo, the functional result of this elevated number of circulating EVs is technically challenging to study. However, the high prevalence of CVDs in the clinic means it is one of the easier areas to obtain rapid and thorough clinical samples.

Vascular dysfunction, in particular activation of endothelial cells, is a hallmark event in atherosclerosis with a phenotypical switch from a quiescent, non‐adherent surface to one that is pro‐inflammatory, pro‐thrombotic and adhesive to circulating immune cells. Endothelial cell‐derived EVs are characterised by a panel of proteins that are associated with endothelial cell biology and activation, and thus are thought to contribute to pathophysiology. These include PECAM1/CD31, VCAM1/CD106, ICAM‐1/CD54, von Willebrand factor (VWF), vascular endothelial growth factor (VEGF), P‐selectin, E‐selectin, endoglin/CD105, VE‐cadherin/CD144, melanoma cell adhesion molecule (MCAM)/CD144, endothelial cell derived nitric oxide synthase (eNOS) (Akbar et al., 2017; Motta‐Mejia et al., 2017), as well as endothelial cell associated microRNAs: miRNA‐126‐3p and miRNA‐126‐5p (Pérez‐Boza et al., 2018). Although these endothelial cell markers are useful to identify endothelial cell‐derived EVs in complex biological fluids such as plasma, how these markers change under pathological conditions is yet to be fully determined under the large umbrella of CVD. It has been shown that endothelial cell‐derived EVs are elevated in acute myocardial infarction and are enriched for VCAM‐1, miRNA‐126‐3p, and EV‐miRNA‐126‐5p (Akbar et al., 2017), which may contribute to the underlying mechanisms.

## 5 THE RENAL SYSTEM

Initially, urine‐derived EVs (uEVs) were mostly studied as biomarkers for kidney and urologic diseases. The wide availability of urine as a biofluid, and the relative ease of obtaining large quantities, made this a more approachable area than other fields of EV biology, such as the study of EVs’ functional role in disease processes of the kidney. Although, there is evolving evidence for additional roles of EVs in inter‐organ communication; cardiorenal syndrome being a good example (Gonzalez‐Calero et al., 2014). For the purposes of this review, we will think about the system as moving along the nephron, the functional unit of the kidney, from the glomerulus that filters blood, towards the tubules and collecting duct system that regulate the urine composition into the tubular interstitial system. Certainly, EVs are found to be involved in a range of pathologies of intrinsic kidney diseases affecting all of these structures, including glomerular and tubular injury, kidney inflammation and fibrosis and disrupted ion and water channel transport (Gildea et al., 2014; Kahn et al., 2017; X. Liu et al., 2020; Lv et al., 2018).

Intra‐nephron communication is challenging to study, and few in vivo works effectively demonstrate the role of EVs in this part of the kidney. However, intra‐glomerular communication within the blood vessels of the glomerula has been already investigated in vasculitis. This is a particularly aggressive disease causing severe endothelial damage in multiple organs, including the kidney and lung. Kahn et al. (2017) have shown that leukocyte‐derived EVs bearing B1‐kinin receptors are enriched in the plasma of vasculitis patients and dock on to endothelial cells in the glomerulus. This work demonstrated that leukocyte‐derived EVs transferred functional receptors to endothelial cells and promoted kinin‐associated inflammation. This study illustrates the potential bidirectional nature of EV communication between the circulatory and urinary systems and highlights several important questions in the field and a chicken‐and‐egg conundrum. It is not known whether circulating EVs from pathology in other systems are filtered into the urinary tract by the kidneys, or whether circulating EVs *cause* kidney pathology, which in turn results in changes in uEVs.

Within the kidney itself and along the nephron units, EV‐mediated communication between different functional regions is also increasingly being explored. EVs have been shown to contribute to glomerular‐tubular communication and may result in the development of pathology. In the setting of albuminuria, tubular epithelial cells have been shown to produce increased numbers of EVs loaded with chemokine mRNA, and have been found to deliver this cargo to interstitial macrophages (Lv et al., 2018). As with many diseases, increased inflammation often results in oxidative damage to cells. In kidney diseases where this occurs, such as IgA (Y. Wang et al., 2016) and diabetic nephropathies (D. Wang, Jin, et al., 2019), understanding how EVs facilitate tubular interstitial inflammation and fibrosis as a result of proteinuria or albuminuria is key to developing new therapies.

Further down the nephron, the propagation of inflammation between the tubule and the interstitium may also cause significant damage to the kidneys. X. Liu et al. (2020) injected TGF‐β1‐treated tubular cell‐derived EVs into mouse kidneys, which resulted in the aggravation of kidney injury and fibrosis, and which was negated by a Shh signalling inhibitor. Another group demonstrated that EVs generated in vivo from kidney tissue of animals with ischemia reperfusion injury transferred TGF‐β1 to fibroblasts in vitro (Borges et al., 2013). Indeed, experimental ischemia‐reperfusion in EV‐deficient mice alleviated the extent of kidney fibrosis (Guan et al., 2020) demonstrating that EVs are capable of modifying the course of disease in vivo.

In contrast to the aforementioned studies on communication between the vasculature and the glomerulus, the tubules and interstitium, intra‐tubular communication between proximal and distal tubular cells has mostly been studied in vitro. For example, proximal tubule EVs have been shown to decrease sodium channel function in distal tubule cells via nucleic acid transfer (Jella et al., 2016). Whilst this communication is of significant clinical interest, in terms of understanding sodium handling in the healthy kidney and in diseases such as diuretic resistance or nephrotic syndrome, it is understudied in the whole animal so its potential for therapeutic intervention, and the role of EVs therein, is currently untapped (Gildea et al., 2014).

Understanding how EVs contribute to renal pathology, in particularly their role in intra‐nephron communication, will open up mechanistic discoveries for many disease processes in the kidney, from acute glomerular and tubular damage to chronic models of kidney fibrosis. Physiologically relevant in vivo models are clearly needed to study their role in renal pathology, as well as their potential to be reflective of broader pathological changes which might affect circulating EVs.

## 6 REPRODUCTIVE BIOLOGY

As outlined in part 1, there is now evidence for EV signalling at every stage of reproduction (see *Part I: Reproductive Biology*) (Simon et al., 2018). However, as well as a role in normal gynaecological processes, dysfunctional EV signalling has been associated with gynaecological pathology and diseases occurring during pregnancy. These include endometriosis (Khalaj et al., 2019), polycystic ovary syndrome (PCOS) (Amabile et al., 2005), pre‐eclampsia (Han et al., 2020), and gestational diabetes (James‐Allan et al., 2020). For example, an EV‐associated RNA (DENND1A—a candidate PCOS gene) is elevated in the urine of women with PCOS, pointing to a possible novel diagnostic test using EVs (Mcallister et al., 2014). EV miRNAs involved in the regulation of estradiol have also been found to be downregulated, suggesting EVs may also play a role in the initiation of PCOS (Sang et al., 2013). Finally, PCOS is associated with a prothrombotic state. This led two groups to quantify platelet‐derived EVs in plasma; both found a greater abundance in women with PCOS, which may contribute to elevated cardiovascular risk in these patients (Koiou et al., 2013; Willis et al., 2014). In addition, Khalaj et al. (2019) identified EVs in the plasma and peritoneal fluid of endometriosis patients which were enriched for miRNA that have previously been associated with disease pathophysiology. Collectively, these findings suggest that endometriosis‐associated EVs carry unique cargo and contribute to disease pathophysiology by influencing inflammation, angiogenesis, and proliferation within the endometriotic lesion microenvironment. Together, these data demonstrate the importance of studying EVs in clinical samples where there is significant potential to establish hitherto understudied mechanisms of disease.

EVs have also been studied in some of the most significant causes of mortality during pregnancy. Pre‐eclampsia (PE) is a disease unique to pregnancy which remains a leading cause of maternal and foetal morbidity and mortality worldwide (Steegers et al., 2010), and is one of the disease states in which EVs have been most studied in clinical samples. However, the bulk of work has been in vitro or ex vivo, using syncytiotrophoblast‐derived extracellular vesicles (STB‐EVs) obtained from cell culture or placental explants of laboratory models of PE, or using STB‐EVs obtained from perfused ex vivo placentas from women with PE. The first publication implicating STB‐EVs in the pathophysiology of PE in 1998 demonstrated higher levels of STB‐EV circulating in the peripheral plasma of women with PE (Knight et al., 1998). in vitro models of pre‐eclampsia have demonstrated reduced abundance of 3’ GAPDH amplicons under hypoxia/reoxygenation conditions and differential expression of miRNAs (Cronqvist et al., 2017; Rusterholz et al., 2007; Wei et al., 2017). Excitingly, in vivo studies are starting to yield results; one group found increased placental EVs in pregnant women with PE compared to those without, which induced hypertension when injected into non‐pregnant mice (Han et al., 2020). Another group reported that a combination of 12 miRNAs isolated from STB‐EVs in peripheral plasma could be used to distinguish normal from PE pregnancies, suggesting that these nucleic acids are involved in pathophysiology.

Finally, normal pregnancy represents a state of relative physiological insulin resistance. In some women the insulin resistance becomes pathological, resulting in a disease called gestational diabetes mellitus (GDM). Similar to findings in preeclampsia, groups have reported that total EV load was increased in the maternal plasma of GDM pregnancies compared to normal pregnancies (James‐Allan et al., 2020; J. Liu et al., 2018; Salomon et al., 2016). Critically, adoptive transfer of plasma‐derived EVs from women with GDM, but not from women without GDM, induced glucose intolerance in non‐pregnant mice. Together these data demonstrate the strong role EVs play in maintaining reproductive health and the progression of a successful and non‐pathological pregnancy. The development of rapid, on‐the‐ground diagnostic tests using EVs is likely to be key in the field of obstetrics and as such research in whole animals and clinical patients will be important in the coming years.

## 7 CENTRAL NERVOUS SYSTEM (CNS) PATHOLOGY

Pathology in the CNS can be broadly divided into two categories; acute injuries, such as stroke and trauma, and chronic neurodegenerative diseases, such as Parkinson's. In acute CNS injury there is damage to multiple cell types. Trauma, for example, causes rapid mechanical damage to the vasculature, neurons and glia, resulting in widespread cell death as well as the release of damage‐associated molecular patterns. However, in neurodegenerative disease, the original pathology is often limited to single cell types, before becoming more widespread. In Parkinson's, the disease is ostensibly caused by death of dopaminergic neurons in the substantia nigra, but is later characterized by widespread neuronal and glial pathology. Therefore, the role EV signalling plays in these pathologies is likely to dependent on the specifics of the disease.

In acute CNS injuries such as stroke and TBI, there are two phases; the primary injury is defined by the mechanical insult and is largely considered irreversible. Whilst there are strategies to reduce the onset of stroke, the primary injury in trauma is often unavoidable and, in most cases, the non‐resolving inflammation that follows is where intervention becomes key. Resident cells of the CNS can release EVs, which contribute to additional damage during this secondary phase of injury. Kumar et al. (2017) showed that there is an increase in EV release from microglial cells after TBI, which induced neuroinflammation when injected into an uninjured brain. In addition, Rong et al. (2018) reported that intrastriatal injection of brain‐derived EVs activated microglia and induced the release of pro‐inflammatory mediators. Thus, EVs can act locally to exacerbate central inflammation after the initial injury.

The secondary phase of injury is not restricted to the CNS. Activation of the peripheral immune system is also involved; indeed, an acute phase response occurs after most CNS injuries. Damage‐associated proteins attract and activate immune cells, such as macrophages and neutrophils, which migrate to the site of injury in order to start repair, but often cause more damage by releasing reactive oxygen species and associated molecules that are normally designed for killing bacteria (Anthony & Couch, 2014). However, the communication pathways between the injured CNS and components of the immune system remain relatively unknown, and EVs provide a promising, novel route for interaction (Anthony & Couch, 2014; Yates et al., 2019). Indeed, rats with an activated CNS immune system have been shown to have more circulating EVs than controls (Couch, Akbar, Roodselaar, et al., 2017). The authors demonstrated the ability of these EVs to induce systemic inflammation in a naïve animal, suggesting that one aspect of their function may be to facilitate communication between the brain and the peripheral immune system. Dickens *et al*., used a similar mouse model of CNS inflammation to demonstrate that EVs derived from astrocytes may play a key role in this process (Dickens et al., 2017). Other studies have demonstrated increases and changes in EVs after stroke (Couch, Akbar, Davis et al., 2017) and TBI (Gill et al., 2018; Hazelton et al., 2018), showing similar activation of the immune system (Couch, Akbar, Davis, et al., 2017; Hazelton et al., 2018), suggesting that EVs may be responsible for brain‐immune communication.

MS sits at the halfway point between chronic neurological disease, and acute injury. Whilst during periods of remission, patients may be able to function normally, during relapses, patients exhibit significant CNS lesions resulting in profound neurological deficits, manifesting in a multitude of problems including visual disturbances and motor deficits. Furlan's group have demonstrated the presence of myeloid+ EVs as potential markers of episodes of neuroinflammation, such as those occurring during an MS relapse (Verderio et al., 2012), and a number of groups have demonstrated increases in circulating EVs in MS patients (Mallardi et al., 2018; Minagar et al., 2001; Sáenz‐Cuesta et al., 2014). Verderio et al. (2012) have demonstrated that EVs are an effective marker for neuroinflammation, by revealing an increase in myeloid‐derived EVs in MS patient cerebrospinal fluid (CSF), which was dependent on patient status (stable vs. acute MS) and correlated with the number of lesions. This suggests they might be used as a way of determining remission and clinical efficacy of novel drugs. Interestingly, the authors also showed that intracranial injection of microglia/macrophage‐derived EVs exacerbated inflammation in the EAE model of MS, whilst A‐SMase KO mice, with reduced EV release, were resistant to EAE, indicating a functional role for EVs in MS pathology.

In chronic diseases such as Parkinson's and Alzheimer's, the pathology usually takes the form of slow degeneration of a subtype of neurons over a number of years, and studies have shown that EVs can propagate these diseases. Sproviero et al. (2021) showed that it was possible to use the miRNA profile of EVs from patients with neurodegenerative disease to not only distinguish them from healthy controls, but also to establish a disease‐specific miRNA profile. In a more specific example, levels of alpha synuclein in neuronal EVs from patients with Parkinson's were used to distinguish them from patients with multiple system atrophy, a distinction which is challenging to make in the clinic in the very early stages of these diseases (C. Jiang et al., 2021). These data show that EVs are phenotypically altered in CNS disease states, which may drive the underlying mechanism. Indeed, pathological amyloid has been found to be enriched in circulating EVs from psychiatric patients, suggesting a contribution to the cognitive deficits in some of these diseases (E. E. Lee et al., 2020). In neurodegeneration, the aggregation of misfolded proteins such as amyloid underlies many diseases and EVs have been suggested as a vector for the propagation of these misfolded proteins. For example, in a mouse model of Alzheimer's disease the propagation of tau fibrils has been shown to be mediated by EVs (Iba et al., 2013) and similar results have been found in mouse models of aging (Ruan et al., 2021). More recent work has demonstrated that EVs derived from familial Alzheimer's IPSC cells are capable of inducing tau pathology in naïve mice (Aulston et al., 2019). In Parkinson's patients, EVs have been suggested as a nexus for alpha synuclein aggregation, where they also have the potential to be used as diagnostic tools for stratifying patients (Alvarez‐Erviti et al., 2011; Stuendl et al., 2016). Whilst there are an increasing number of studies investigating the potential for EVs to play a role in these diseases, mechanistic studies still largely remain in vitro (Eitan et al., 2016; Nikitidou et al., 2017; Sardar Sinha et al., 2018).

## 8 MUSCULOSKELETAL PATHOLOGY

Pathology within the musculoskeletal system is particularly detrimental to the whole organism since it provides the framework of the body. These pathological processes also often underlie pain and lack of mobility, which are significant burdens on the healthcare system. By understanding more about how EVs are involved in the genesis and potentiation of pathology within the system, we may be able to develop novel interventional strategies (Figures 1 and 2).

### 8.1 Skeletal muscle

Cachexia syndrome is a complex disease characterized by loss of skeletal muscle and adipose tissue, under pathological circumstances. This syndrome is frequently caused by cancer, but may also be a result of chronic infections, heart failure and other diseases. Not all types of cancer induce cachexia syndrome, but between 50% and 80% of cancer patients lose weight, both muscle and fat, and these patients often have increased levels of circulating EVs (Chitti et al., 2018). Two common markers found in EVs, HSP70 and HSP90, are released at high levels in tumour‐EVs and have been shown to actively induce muscle wasting (G. Zhang, Liu, et al., 2017). Therefore, circulating EVs may play an active role in this pathology.

Much of the research on EVs in muscle pathology is focused on Duchenne muscular dystrophy (DMD), particularly in terms of biomarkers and therapeutics, rather than pathophysiology. Despite significant scientific advancements, DMD remains incurable and diagnosis is based on analysis of muscle biopsy tissue, an invasive and sometimes painful procedure. Research on EV biology has suggested that EVs may be used to aid early DMD diagnosis, as well as our understanding of the mechanistic underpinnings of the disease (Coenen‐Stass et al., 2017), but their contents and function are still under investigation (Rogers et al., 2019). A recent paper from Gartz et al. (2020), showed that GW4869 treatment to reduce exosome release (by neutral sphingomyelinase [nSMase] inhibition) in mdx mice (a popular model of DMD) was protective against cardiac stress, which authors attributed to miR cargo. In line with this, Matsuzaka et al. (2020) showed that ablation of nSMase2/Smpd3 gene in mice with a mdx background decreased muscle inflammation and improved functionality.

### 8.2 Bone

Pathology within the skeletal system can cover any number of diseases, from osteological tumours to chondrocytic disease and inflammation. The underlying disease is likely to dictate the EV populations within the circulation, and thus their eventual effects on downstream cells and organ systems. For example, one of the major diseases associated with the skeletal system is arthritis, inflammation of the joints. An early paper from H.‐G. Zhang et al. (2006) showed that synovial fibroblasts from patients of RA produced small EVs containing the inflammatory protein TNF‐alpha, but this was not present in the EVs of osteoarthritis (OA) patients, further demonstrating that the EVs from RA patients stimulated NFkB production. A more recent study of small EVs collected from the synovial fluid of men and women with OA showed that their EVs reduce metabolic activity of the cell population, as an indicator of cell viability (Kolhe et al., 2017). Interestingly, when they profiled the miRNA content of the EVs they showed enrichment for targeting of sex‐specific signalling pathways, a factor which is key in a disease that is more prevalent in women These studies highlight the importance of combining clinical data with pre‐clinical research. Although, it must be noted that the pre‐clinical community often uses young, healthy male mice, which may not necessarily be reflective of the clinical population.

## 9 THE GUT MICROBIOME

As explored in Part I, microorganisms of the gut microbiome release EVs, termed Outer Membrane Vesicles (OMVs), which can act both locally and systemically to maintain homeostasis. However, OMVs can also be released by harmful bacteria and contribute to driving disease. *Helicobacter pylori* (Hp) is a Gram‐negative proteobacteria whose lingering infection is one of the most relevant drivers of chronic inflammation and the development of gastric cancer (Ishaq & Nunn, 2015). As a common dormant infection in man (Eusebi et al., 2014), Hp provides a useful model system for investigating the interaction between bacterial EVs and the human host. Hp OMVs have been shown to carry the virulence genes (CagA and VacA) of the parent bacterium, and the oral administration of Hp‐OMVs in mice has shown that they can stimulate the production of a number of immunomodulatory cytokines in macrophages and gastric epithelial cells (Choi et al., 2017). Importantly, Cy7 labelled Hp‐OMVs (but not Hp per se) were also able to infiltrate the gastric epithelium, where they could still be detected 24 h after injection (Choi et al., 2017). In this sense, it seems that Hp‐derived OMVs may induce inflammation and their chronic presence may contribute to the development of complications such as gastric cancer (Choi et al., 2017). Further work has demonstrated EV‐mediated communication between Hp‐infected cancerous cells and macrophages (Che et al., 2018). In this work, Che et al., showed that a phosphorylated active growth factor isoform was enriched in EVs released from Hp‐infected gastric cancer cells, which were delivered to, and internalized by, macrophages. The internalization of these growth factor‐enriched vesicles had an apparent role in educating the macrophages towards a pro‐tumorigenic phenotype, including increased secretion of IL‐1β, that could promote tumour growth and progression in vivo. Together, these publications demonstrate an important role for Hp‐derived OMVs in the establishment of gastric cancer. The microbiota is incredibly diverse, in terms of species and strains, and it is therefore not unreasonable to assume that other microbiota‐derived OMVs play key roles in other disease processes.

In addition to local effects, the gut also maintains significant communication links with other parts of the body. The gut‐brain axis (GBA) is emerging as an important player in psychiatric diseases (Kelly et al., 2015). Studies have demonstrated that increased GBA permeability during childhood may result in autism spectrum disorders (Fowlie et al., 2018), with microbiota by‐products, including EVs, getting into the circulation and interfering with normal development (Haas‐Neill & Forsythe, 2020). Certainly, Lee et al. have demonstrated that it is possible to use bacteria‐derived EVs in the urine to profile the microbiome in autism spectrum disorders, with the aim of prophylactically treating toddlers with pre‐ and pro‐biotics to combat an differences in microbiotal diversity (Y. Lee et al., 2017). Because the microbiome is known to play a role in a number of mental health disorders including depression and anxiety (Skonieczna‐Żydecka et al., 2018), EVs derived from gut microorganisms may contribute, either directly or indirectly, to the underlying pathophysiology.

## 10 CANCER

Cell growth and division are processes active throughout life, and, in normal physiology, are strictly regulated to preserve tissue architecture. Dysregulation of cell proliferation can lead to growth diseases. These include aplasia and hypoplasia, hyperplastic diseases, psoriasis, gigantism, tuberous sclerosis, hamartoma, and neoplasia. The role of EVs has been studied some of these diseases (Cheung et al., 2016; Grapp et al., 2013; M. Jiang et al., 2019; Patel et al., 2015; Z. Wang, Zhu, et al., 2019) but the majority of work recently has focused on their role in cancer (Carretero‐González et al., 2018; Rajagopal & Harikumar, 2018). When considering the role EVs might play in the basic regulation of the cell cycle and cell growth there is a distinct lack of true in vivo experiments, in which EVs collected in vivo are also applied to in vivo models of cell cycle progression or inhibition. This is likely due to the inherent difficulties in enriching uncontaminated EVs from biofluids which are highly susceptible to the influence of pre‐analytical variables. Advancements in these enrichment techniques, in combination with a relatively recent surge in the use of BrdU to detect new DNA synthesis in vivo, will hopefully pave the way for such experiments to be performed in the not‐so‐distant future. Such experiments will enable us to develop significant insights into the potential for EVs to play roles in the initiation and maintenance of an oncological state. Despite this lack of fundamental mechanistic understanding, the field of EVs in cancer diagnostics and therapy has exploded recently (Shehzad et al., 2021) and is a fertile ground for the development of novel in vivo models to study cell growth. By combining the mine of information on EV biomarkers in cancer from the clinic with in vivo models of disease we may be able to determine their function in pathology, something which is currently lacking in the field.

Carcinogenesis is a multistep process that begins when a normal cell achieves a selective growth advantage through the accumulation of multiple (epi)genetic alterations (Knudson, 2001). In the last two decades, EVs have been shown to play decisive roles in tumour development by transferring oncogenes, regulating tumour‐stroma interactions, developing the pre‐metastatic niche and encouraging angiogenesis (Costa‐Silva et al., 2015; Fong et al., 2015). Whilst there are significant physiological changes which occur during cancer progression, such as alterations in metabolism, these have not yet been studied in vivo in the context of EV signalling. Therefore, this section will focus on the areas above, where the majority of in vivo EV research has focused.

For oncogene transfer, there is significant evidence demonstrating that oncogenic proteins within cancer cell‐secreted EVs can be transferred to recipient cells, conferring an aggressively cancerous phenotype. Soldevilla et al. (2014) have shown that EVs from colon cancer cells contain, and can transfer, an active form of the NH2‐terminally truncated p73 (DNp73) oncoprotein, and thus promote tumour growth when injected in athymic nude mice. Similarly, astrocytoma cells overexpressing the oncogenic epidermal growth factor receptor variant III (EGFRvIII) are able to enhance the aggressiveness of glioma cells by transferring EGFRvIII through EVs (Al‐Nedawi et al., 2008). More recently, it has been shown that apoptotic cells within glioblastoma tumours release EVs which promote cell proliferation and aggressive migratory characteristics in adjacent tumour cells (Pavlyukov et al., 2018). These apoptotic EVs exert their function by transferring RBM11, an RNA binding protein, to surviving recipient glioma cells where it alters RNA splicing towards the expression of the more oncogenic isoforms of MDM4 and cyclin D Lindoso *et al*. showed that cancer stem cells also release EVs capable of driving a pro‐tumourigenic phenotype in mesenchymal stromal cells in renal cancer (Lindoso et al., 2015). Together these data suggest that one of the functions of EVs in cancer is to promote the further growth of the cells within the tumour.

In addition to the transfer of proliferative capacity, EVs have the potential to alter several other aspects of tumour biology. Tumours are more than masses of uncontrollably proliferating cells. There is a constant bidirectional crosstalk between tumour cells and neighbouring stromal cells, and EVs have been shown to be key to this communication. For example, prostatic cancer‐derived EVs can transform normal fibroblasts to myofibroblasts or cancer‐associated fibroblasts (CAFs), known to promote cancer invasion and metastasis (Abdouh et al., 2019; Kong et al., 2019; Webber et al., 2015). These activated CAFs secrete pro‐inflammatory cytokines such as IL‐6 and IL‐8, further priming tissue potential for cancer metastasis (Fang et al., 2018). In Hodgkin's lymphoma, tumour EVs regulate the tumour microenvironment by stimulating fibroblasts to release tumour promoting cytokines (Dörsam et al., 2018). Moreover, in a model of gastric cancer, tumour‐derived EVs were found to be enriched with the same miRNAs that, when directly transfected, induced expression of CXCL1 and CXCL8 in CAFs, indicating the potential for the EV miRNAs to induce the same effect (Naito et al., 2019). Clinically, expression of these chemokines in CAFs correlated with poorer patient survival (Naito et al., 2019), once again demonstrating the importance of studying clinical markers in pre‐clinical models of disease. As well as cancer cells signalling to the stroma, stromal cells have also been reported to communicate with tumour cells via EVs. NOTCH‐MYC signalling in breast cancer fibroblasts results in the accumulation of the RN7SL1 RNA, without its protein counterpart, in EVs (Nabet et al., 2017). These EVs then trigger anti‐viral signalling in neighbouring breast cancer cells leading to a local inflammatory response and subsequent tumour progression. These data highlight the lifetime interplay between tumour and stromal cells and the immune system and the key role EVs play at this junction.

A key aspect of the communication pathway between tumour cells and stromal cells is the initiation of metastasis. Metastasis is a milestone in cancer progression, which dramatically increases mortality. It is a complex process that can be divided into angiogenesis and invasion of surrounding tissues, entry, circulation and exit from the vascular system and colonization of the distal site (Brooks et al., 2010; Nicolson, 2015). During this lengthy escape, cancer cells require the ancillary recruitment of other cells in the body to pass the physical barriers (e.g., endothelium) or even to pave the way (pre‐metastatic niche formation) towards the secondary site. EVs have emerged as pivotal players in all steps of the metastatic cascade (Becker et al., 2016; Meehan & Vella, 2016). For example, a plethora of studies have shown that tumour‐derived EVs induce angiogenesis through different mechanisms (Al‐Nedawi et al., 2009; Dörsam et al., 2018; Grange et al., 2011; Hsu et al., 2017; Janowska‐Wieczorek et al., 2005; Lindoso et al., 2015; Lopatina et al., 2018; Lu et al., 2018). Breast cancer cells have been shown to release Annexin II‐containing EVs that initiate angiogenesis by stimulation of the tissue plasminogen activator (Maji et al., 2017). Hypoxic tumour‐derived EVs favour angiogenesis in lung and myeloid carcinoma respectively, which may involve miR‐23a and miR‐135b cargo (Hsu et al., 2017; Umezu et al., 2014).

In addition to angiogenesis, EVs have emerged as a new component in the regulation and development of the tumour microenvironment, supporting the formation of the pre‐metastatic niche and organotropic metastasis (Costa‐Silva et al., 2015; Deng et al., 2017; Fabbri et al., 2012; Hoshino et al., 2015; Keklikoglou et al., 2019; Peinado et al., 2012; H. Zhang, Deng, et al., 2017; L. Zhang et al., 2015). EV Annexin II, as well as promoting angiogenesis, is also involved in priming the metastatic niche for breast cancer cells migrating to brain through the activation of STAT3 and p38‐NFkB pathways (Maji et al., 2017). Further studies on breast cancer metastasis have demonstrated that miRNAs in EVs lead to modulation of actin dynamics in order to induce the destruction of the blood‐brain barrier (Tominaga et al., 2015). Melanoma and breast cancer cells metastasising to the brain take up EVs from astrocytes carrying miR‐19a which, in turn, downregulates PTEN in recipient cells and results in rapid cancer outgrowth at the secondary site (L. Zhang et al., 2015). Wysoczynski and Ratajczak (2009) demonstrated that exposing stromal cells to EVs from tumour cells resulted in the stromal cells releasing factors, which encouraged the motility and metastatic capacity of lung cancer cells in vivo (Challagundla et al., 2015). Elsewhere in the body, pancreatic cancer derived EVs trigger signalling in Kupffer cells (stellate liver macrophages) resulting in extracellular matrix remodelling (Costa‐Silva et al., 2015). This results in fibronectin accumulation and macrophage recruitment to the liver, generating a metastatic niche within the liver. Gastric cancer is also known to metastasise to the liver, a process mediated by EGFR‐containing EVs shed by gastric tumour cells (H. Zhang, Deng, et al., 2017).

Taken together, EVs have been shown to play important roles in all aspects of tumour biology. They are capable of transferring proliferative capacity in a paracrine manner via proteins, growth factors and miRNAs, they have been shown to act as communication vectors between stroma and tumour, working to increase angiogenesis at the tumour site and they have been shown to prime the metastatic niche for the migration of tumour cells around the body. Whilst no single component has been suggested as master regulator of these processes, and they vary between cancers, EVs still remain an important source of potential biomarkers and a viable drug target in the field of oncology.

## 11 CONCLUSION

It is clear from the studies included in this review that, although EV‐mediated signalling plays a significant role in immune dysfunction and driving disease dissemination, it is not due to the impairment of the signalling mechanism itself, but rather its appropriation in unfavourable conditions. Therefore, EVs can be considered neither good nor bad, as their effect is dependent on the physiological state of the cell. Instead, they can be considered innocent bystanders that are manipulated. This concept provides some relief as EV signalling may then be manipulated therapeutically to benefit the host and treat diseases and disorders, for example, EVs released from stem‐cells are of particular interest (Webb et al., 2018). However, to progress the field, we must seek to investigate the mechanisms underlying EV signalling in vivo wherever possible using populations of EVs with well‐defined characteristics. By doing so we may be able to draw more accurate conclusions regarding the function of mixed populations of EVs in both normal physiology and during pathology.

## CONFLICT OF INTEREST

Ryan Pink is currently CEO of MetaGuideX, an exosome diagnostics company.
